# Comparison of Three Gestational Weight Gain Guidelines Under Use in Latin America

**DOI:** 10.3389/fped.2021.744760

**Published:** 2021-10-13

**Authors:** Francisco Mardones, Pedro Rosso, Álvaro Erazo, Marcelo Farías

**Affiliations:** ^1^Centro Latino Americano de Estudios Económicos y Sociales (Latin American Center for Economic and Social Studies), Pontificia Universidad Católica de Chile, Santiago, Chile; ^2^Department of Pediatrics, School of Medicine, Pontificia Universidad Católica de Chile, Santiago, Chile; ^3^Department of Obstetrics and Gynecology, School of Medicine, Pontificia Universidad Católica de Chile, Santiago, Chile

**Keywords:** guidelines, Latin America, gestational, weight, gain

## Abstract

Presently, three guidelines are used in Latin America to assess adequacy of maternal body mass index (BMI) during pregnancy: (1) the chart proposed by the Institute of Medicine of the United States (IOM), (2) the Rosso-Mardones Chart (RM), and (3) a modified RM chart proposed by Atalah et al. (AEA). The aim of the present review was to explore available information on the sensitivity, specificity, and both positive (PPV) and negative predictive values (NPV) of these charts to detect women at risk of delivering babies with the following signs of abnormal fetal growth: (a) length at birth (BL) <50 cm; (b) birth weight (BW) <3,000 g; and (c) BW ≥ 4,000 or 4,250 g. Data from studies conducted in large samples of Chilean and Uruguayan women indicate that the RM chart has the greatest sensitivity to identify at risk cases. However, predictive values were similar for the three charts. Thus, the use of the RM chart should be preferred. The main limitation for using the IOM weight gain recommendations in Latin American women stems from the fact that their average height is approximately 20 cm lower than US women.

## Introduction

Over the last three decades, in most Western countries the proportion of overweight and obese women of reproductive age [body mass index (BMI) 25.0 kg/m^2^ or more] underwent a substantial increase (1). In Chile the proportion of obese women (BMI 30.0 kg/m^2^ or more) was 11% in 1988 and 37% in 2017 (2, 3). A similar trend has been reported in the USA (4, 5). One of the factors contributing to this obesity epidemic would be an excessive weight gain during successive pregnancies (4), an observation that highlights the importance of monitoring this aspect during pregnancy.

Body weight gain in a gravida reflects both maternal physiological adaptations and growth of the fetus, placenta, and accumulation of amniotic fluid. The main maternal adaptations include blood volume expansion and body fat accumulation (6).

Women who are either overweight or underweight at conception are at risk of maternal-fetal complications (1, 6, 7). In obese women pregnancy complications include hypertension, gestational diabetes, dystopian childbirth, and fetal macrosomia. In women with low weight/height the main complication is fetal growth retardation. The risk of these complications increases if obese women gain an excessive amount of weight and thin women gain little weight during their pregnancies. In both situations the offspring have a higher incidence of metabolic syndrome later in life. Consequently, assessment of maternal weight/height adequacy in early pregnancy and monitoring weight gain during pregnancy are considered key aspects of maternal and child health care (1, 6–8).

Despite consensus regarding the importance of maternal gestational weight gain an agreement has yet to be reached concerning its quantitative aspects. Consequently, a universally used instrument (chart) of desirable weight gain for a given maternal weight/height at conception is lacking (9). Currently, most countries in the Northern Hemisphere use the guidelines of the United States Institute of Medicine (IOM) while most Latin American countries use the Rosso-Mardones instrument (RM) or its modification authored by Atalah et al. (AEA) (9–12). The main objective of this mini review was to compare the accuracy of these instruments to identify pregnancies at risk of fetal growth alterations in Latin American women.

## Guidelines for Anthropometric Assessment of Maternal Nutritional Status

Available guidelines for adequacy of maternal weight/height use the Quetelet Index, also known as BMI (8) and individual targets for weight gains during pregnancy.

### United States Institute of Medicine Guidelines

These guidelines were developed in 1990, but the cut-off points for appropriate BMI were modified in 2009 following World Health Organization (WHO) recommendations for adult non-pregnant women (8, 10, 13). Those BMI cut-offs are calculated pre-conceptionally by asking the women about their usual weight and also measuring their height. The various categories of maternal nutritional status are as follows: (a) Low weight: BMI <18.5 kg/m^2^; (b) Normal: BMI 18.5–24.9 kg/m^2^; (c) Overweight: BMI 25–29 kg/m^2^; and (d) Obese: BMI ≥30 kg/m^2^ (8, 10). For these categories, the IOM recommends the following weight gains during pregnancy: (a) Women with “low weight” should gain 12.5–18.0 kg. (b) Women with “normal” BMI should gain 11.5–16 kg. (c) Overweight women should gain 7.0–11.5 kg. (d) Obese women should gain 5.0–9.0 kg. The 2009 IOM guidelines considered for the first time the outcomes of both mother and child during and after delivery and the trade-offs between them (4); the recommended weight gain ranges were those most consistently associated with good outcomes, including reduced post-partum weight retention.

WHO's weight/height ratio categories are based on the relationship between BMI and mortality or life expectancy in adult non-pregnant women. The lower risk of mortality is associated with a BMI between 18.5 and 24.9 kg/m^2^ (8). Thus, *strictu senso* they do not represent “normalcy” in a pregnancy situation. Institute of Medicine's recommendations are based on measurements made in a racially mixed general US population. This is certainly advantageous for a worldwide use, but average height of US women is 176 cm (10, 13). Thus, it is significantly higher than women living in the Southern Hemisphere. For example, average height of adult women in Ecuador is 152 cm and in Chile 156 cm (14, 15). This aspect is relevant for two reasons. Firstly, because maternal height is directly and significantly associated with the offspring birth weight (BW) (1, 6, 7, 16). Additionally, because desirable gestational weight gain might differ greatly according with maternal height. For example, a mother who is 140 cm tall and has a “normal” BMI at the beginning of her pregnancy must gain only 10.8 kg to reach term with a normal BMI of 27.6 kg/m^2^. However, a woman who measures 180 cm and has a “normal” BMI when she becomes pregnant, to reach at term a normal BMI of 27.6 kg/m^2^ must gain 18.1 kg. These cases, who are not uncommon, illustrate the importance of establishing weight gains proportional to maternal height.

The IOM guidelines have proved their usefulness in developed countries. For example, a recent systematic review and meta-analysis of more than 1 million pregnant women, showed that gestational weight gain greater than or less than guideline recommendations, compared with weight gain within recommended levels, was associated with higher risk of adverse maternal and infant outcomes (17). From 23 selected studies, 18 were retrospective, and five were prospective. Ten were from the United States, eight were from Asia (four from China, two from Korea, and one each from Taiwan and Japan), and five were from Europe (one each from Norway, Belgium, Italy, Denmark, and Sweden). Sample sizes ranged from 1,034 to 570,672 women. However, in a large German population of overweight and obese mothers those who gained weight within the IOM recommendations had a lower incidence of pre-eclampsia and fewer non-elective cesarean deliveries, but higher risk for gestational diabetes, small-for-gestational-age birth, pre-term delivery, and perinatal mortality (18). Thus, IOM recommendations would be adequate for underweight and normal weight mothers, but different guidelines or thresholds might be more appropriate for overweight and obese ones (1).

### Rosso-Mardones Guidelines

**Figure 1:**
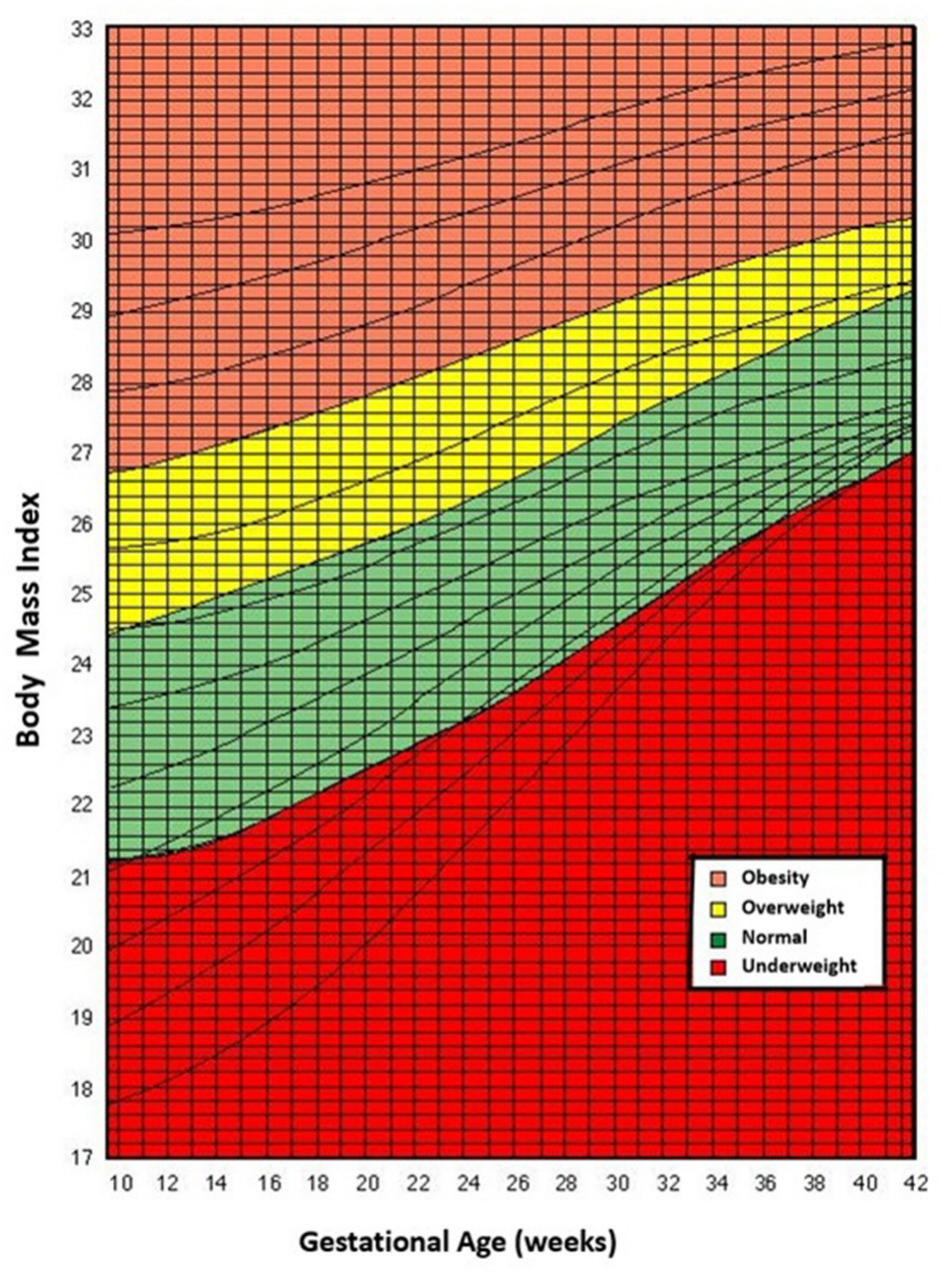
Rosso-Mardones chart.

These guidelines were based on a study conducted on the early 1980s in 1,745 healthy Chilean women who had uneventful pregnancies and full-term deliveries. This group was representative of a Chilean general population. Average height of the study subjects was 154 cm. Data was used to establish recommendations for the entire range of BMIs beginning in the 10^th^ week of gestation (11) (Figure 1). The recommendations were aimed at an outcome of a baby with a “normal” or “desirable BW,” defined as the average BW of the babies born at term, delivered by healthy women, with normal weight/height at the beginning of pregnancy and weight increases considered appropriate for their heights.

The area of “normality” of BMI at the end of pregnancy was defined as one that favors the occurrence of “desirable BWs” (11). We deemed this BMI the maternal “critical body mass” for normal Chilean women, since it would allow optimal fetal growth, as determined by genetic and epigenetic factors. The diagnosis of “low weight” and maternal “overweight” corresponds to mothers whose BMI is below and above this “critical body mass.” Body mass index cut-offs values of the RM chart are presented in Table 1 at the beginning and at the end of pregnancy.

**Table 1:** Body mass index (BMI) kg/m^2^ cut-off points of the RM chart and the AEA chart for the nutritional classification of women at the beginning and at the end of pregnancy (11, 12).

**BMI kg/m^**2**^ cut-offs points**	**RM chart**	**AEA chart**
**Week 10**		
Underweight	<21.15	<20.2
Normal	21.15–24.49	20.2–25.2
Overweight	24.50–26.73	25.3–30.2
Obese	>26.73	>30.2
**Week 40**		
Underweight	<26.55	<25.0
Normal	26.55–28.90	25.0–29
Overweight	28.91–30.03	29.1–33.1
Obese	>30.03	>33.1

### Atalah et al. Guidelines

The Chilean Ministry of Health used the RM Chart from 1986 to 2004. In 2005 it was replaced by a modified version proposed by Atalah et al. (12). Table 1 presents BMI cut-offs of this chart in comparison with the RM chart; there is a clear increment of the normal area in the AEA chart at the beginning and at the end of pregnancy. In contrast with the RM chart, the AEA chart is based on the categories of BMI defined by the IOM. Consequently, the AEA chart classifies as “normal” a percentage of women that according to the RM chart should have been classified as either “underweight” or “overweight.” Consequently, from 2005 on, the use of AEA Guidelines meant a marked apparent decrease in the number of pregnant women with “low weight” and “overweight” registered by the Chilean Ministry of Health. Accordingly, these women did not receive the nutritional counseling and support aimed at risk women.

### Diagnostic Ability of the Three Instruments

The consequences of underestimation and overestimation of pregnancies at nutritional risk by the various guidelines previously analyzed have been investigated by comparing the sensitivity, specificity, positive predictive values (PPV), and negative predictive values (NPV) of the IOM, RM, and AEA guidelines, in pregnant populations of Chile and Uruguay (19–21). In this case the “disease” was defined as the presence of inadequate fetal growth: (a) length at birth (BL) <50 cm; (b) BW <3,000 g; and (c) BW ≥4,000 or 4,250 g.

Sensitivity indicates the proportion of correctly diagnosed cases with the disease and specificity the proportion of healthy cases correctly diagnosed. The PPV indicates the probability that the patient has the disease and the NPV allows knowing the probability that the patient does not have the disease.

The first of these studies was carried out using data obtained in 11,465 healthy Chilean women with singleton pregnancies and gestational age of delivery 39–41 weeks (19). A total sample of 27,613 women with anthropometric and health information was recruited in that study and a subsample of 11,466 healthy pregnant women was selected from the total sample as a control to ascertain the effect of maternal nutritional status on the newborns growth excluding the effect of other factors. The adequacy of the maternal BMI at the beginning of pregnancy was diagnosed by applying the cut-off points of the AEA and RM charts. The comparison of the RM and AEA charts was based on the proportion of children with inadequate fetal growth (according to the indicators described above) in the categories of mothers with low weight and obesity detected by these charts. The RM chart showed higher sensitivity values for diagnosing mothers at risk than the AEA chart, although the predictive values were similar (Table 2). Despite this similarity in the proportions of PPV and NPV, the PPV differs in the number of subjects diagnosed with impaired fetal growth investigated by the RM chart, since it reaches almost double those investigated by the other chart, a situation that is repeated in the two studies discussed below (20, 21).

**Table 2:** Sensitivity, specificity, and positive and negative predictive values for RM and AEA charts corresponding to each target event^*^ (BL <50 cm; BW < 3,000g and BW > 4,250 g) in the total sample (*n* = 27,613) (19).

**Target event**	**Chart**	**Sensitivity**	**Specificity**	**PPV**	**NPV**
BL < 50 cm	RM	0.17	0.87	0.54	0.54
	AEA	0.10	0.93	0.56	0.53
BW < 3,000 g	RM	0.19	0.86	0.28	0.79
	AEA	0.12	0.92	0.29	0.79
BW > 4,250 g	RM	0.73	0.51	0.05	0.98
	AEA	0.65	0.59	0.05	0.98

* *BL, birth length; BW, birth weight; PPV: positive predictive value, NPV: negative predictive value*.

The second comparative study of the RM and AEA charts was carried out in Uruguayan women (20). Data from 23,832 healthy pregnant women, with single deliveries and gestational age of delivery between 39 and 41 weeks were used. The adequacy of BMI in early pregnancy was classified using the AEA and RM charts to define nutritional status at the beginning of pregnancy. When comparing the sensitivity, specificity, and PPV and NPV of both patterns to detect women at risk of inadequate fetal growth, the RM chart again showed higher sensitivity values and predictive values similar to the AEA chart.

The third study in this series consisted of a comparison of the IOM and RM charts in the Uruguayan pregnant population of the previous study, using a design, criteria, and definitions similar to those previously described (21). The RM curve showed significantly higher sensitivity values than the IOM criterion. The predictive values of both charts were also similar.

The three studies presented showed lower specificity values for the RM chart. Since in the three studies the more sensitive RM chart had a much higher number of at risk BW and BL cases in the underweight and obese categories, it is preferable to sacrifice the higher specificity of the AEA or the IOM charts and use the RM chart.

## Discussion

A growing body of evidence suggests that both high and low gestational weight gains are independently associated with an increased risk of child obesity (22). Multiple randomized controlled trials have been conducted evaluating the efficacy of lifestyle interventions on gestational weight gain, and while those interventions may alter gestational weight gain, they have not been associated with improvement in perinatal outcomes (23). The revised comparisons of the three guidelines permitted to assess improved perinatal outcomes when using the RM chart. Although the comparisons did not use an experimental design, the compared groups had exactly the same control variables; the comparison of identical cohorts is similar to a randomized controlled trial. Those results reveal the public health importance of using the RM chart in the populations proposed.

Possible additional interventions that might improve the effect of gestational weight gain on perinatal outcomes have been the following: (A) Weight loss during pregnancy because it has been associated with decreased risks of macrosomia and cesarean section; however, given an association with low BW, it is not currently recommended (23). (B) Research supports the need to achieve a healthy weight pre-conceptionally. In some studies, pre-pregnancy BMI is strongly related to health outcomes in mother and offspring, with even stronger effects of pre-pregnancy BMI than of gestational weight gain on key outcomes (24).

As indicated by the results and conclusions of a recent seminar on maternal nutrition (25), this is an area of evolving studies. There are numerous gaps of knowledge and unresolved scientific debates on the nutritional requirements of the pregnant woman and, to complicate matters further, ostensible cultural changes are underway, expressed in aspects such as the age of the first pregnancy, the massive incorporation of women into the workforce, a greater interest in nutrition and “healthy” foods, etc. All of these developments open new possibilities and pose unprecedent challenges for the nutritional care of pregnant women. Significantly, perhaps reflecting the fact that, for many specialists, the issue of nutritional assessment of pregnant women has been resolved, this issue was not included in the previously alluded seminar (25).

However, the available evidence indicates, that maternal nutrition is an area that requires urgent attention and a “fresh look” at the strength of the scientific support for public policies. The IOM Guidelines appears to be suitable for the U.S. population and for other regions where pregnant women have a similar average height. However, they could be improved by introducing specific weight recommendations for pregnant women whose heights considerably deviates from average. Therefore, its use in populations with average heights of pregnant women <160 cm does not seem advisable.

As the Chilean experience shows, seemingly minor changes in the evaluation criteria can cast long shadows in terms of their effects at the population level. A recent publication shows that in Chile the frequencies of babies with BW <3,000 g and birth length <50 cm increased markedly after 2005, coinciding with the replacement of the RM Chart by the AEA Chart (26). Thus, suggesting a causal relationship. The replacement of the RM Guidelines by the AEA Guidelines has meant a decrease capacity to correctly diagnose and treat mothers at risk of having newborns with low weight or excessive BW. This finding has important implications, including the well-known U-shaped relationship of BW with neonatal and infant mortality (27). Both children weighing <3,000 g and body length <50 cm at birth and those with a BW ≥4,250 or ≥4,000 g are at a higher risk of complications and dying than those of normal weight.

In addition, solid scientific evidence supports the possibility that inadequate fetal growth, manifested in a low or excessive BW (macrosomy), is associated with the early origin of chronic diseases of adults, such as obesity, arterial hypertension, and diabetes (28, 29). Hence the importance of early detection and effective treatment of mothers at nutritional risk of inadequate fetal growth. From this standpoint, because of its greater diagnostic sensitivity for mothers at nutritional risk, and because of its ease of use in populations that have an average height lower than the US population, the RM Guidelines offer significant advantages over other guidelines (1, 6).

## Author Contributions

FM and PR contributed to conception and design of the study. AE and MF organized the references of information. All authors contributed to manuscript revision, read, and approved the submitted version.

## Conflict of Interest

The authors declare that the research was conducted in the absence of any commercial or financial relationships that could be construed as a potential conflict of interest.

## Publisher's Note

All claims expressed in this article are solely those of the authors and do not necessarily represent those of their affiliated organizations, or those of the publisher, the editors and the reviewers. Any product that may be evaluated in this article, or claim that may be made by its manufacturer, is not guaranteed or endorsed by the publisher.
